# The importance of end-of-life welfare

**DOI:** 10.1093/af/vfab078

**Published:** 2022-03-17

**Authors:** Heather Browning, Walter Veit

**Affiliations:** 1 London School of Economics and Political Science, Centre for Philosophy of Natural and Social Science, London, UK; 2 University of Sydney, School of History and Philosophy of Science, Sydney, Australia

**Keywords:** animal welfare science, end-of-life welfare, shape of a life, slaughter, transport

**Implications**

• The conditions of transport and slaughter at the end of their lives are a major challenge to the welfare of agricultural animals.

• End-of-life experiences should be of a greater ethical concern than others of similar intensity and duration, due to their position in the animal’s life.

• End-of-life welfare can have both internal importance to the animals and external ethical importance to human decision-makers.

• We should pay extra care to ensure that the conditions during transport and slaughter are as positive as possible.

## Introduction

One of the major challenges to the welfare of animals in agriculture is the conditions of transport and slaughter. Worldwide, over 70 billion animals are slaughtered for agriculture each year, which places this as a particularly significant ethical issue (Sanders, 2020). There is now a large literature on the need for humane animal slaughter (e.g., Mellor and Littin, 2004; RSPCA, 2019; Browning and Veit, 2020c) and work on improving methods of animal slaughter to ensure a painless death, addressing harms such as the possibility of failure of stunning before the slaughter and processing begin (Welty, 2007; Grandin, 2010) and rough treatment during handling within the slaughterhouse (Grandin, 2010). Of additional concern is the period of time consisting of transport from farm to slaughterhouse, which is where most agricultural animals arguably experience the worst parts of their lives, a time of prolonged stress and suffering including heat stress, crowding, dehydration, fear, and pain from transport injury and rough handling. For instance, animals transported long distances can be subjected to temperature extremes, such as sheep exported by ships from Australia, which suffer extreme heat stress during the warmer months (Collins et al., 2018; RSPCA, 2020).

In this paper, we argue that these harms should be paid special attention over other equivalent types of suffering that an animal may experience throughout its lifetime, because of its position at the end of life. Although, as we have argued previously, while no slaughter can ever be considered truly “humane,” where slaughter is to continue, we should pay special attention to ensuring that animals have as positive an experience as possible during this ethically significant period of their lives (Browning and Veit, 2020c). There are two ways in which we can think about the importance of end-of-life welfare, both of which we will consider in this paper. The first is an internal importance to the animals themselves, through impacting their welfare in ways that matter to them. Secondly, end-of-life welfare can also have features that matter ethically, to the human observers and caretakers who are making ethical decisions on their behalf (an external value). While it is true for animals that they can have better or worse welfare—that there are things that matter more or less to them—ethics is a human endeavor, and thus in the end the ethical significance of some feature or event is determined by human evaluations of its importance. While this will in part include its impact on animals, it can also include other features, such as some we will discuss. We will present two primary reasons why end-of-life welfare holds particular significance—considerations of the shape of a life, particularly the lack of future “compensatory” experiences, and the emotional importance of the harm.

Throughout this paper, we adopt a subjective conception of animal welfare, which grounds animal welfare in the affective (positively and negatively valenced) experiences of animals. Because this is one of the main viewpoints in animal welfare science and we have defended it elsewhere in length (Browning, 2020a, 2020b, 2020c; Browning and Veit, 2020b; Veit and Browning, 2021), we will take this view for granted within the scope of this article, though many of our arguments will equally well apply even if one takes an alternative view on animal welfare. This means that when we are judging whether an animal has good or bad welfare, this can be assessed in terms of the number and types of positive and negative subjective experiences that the animal undergoes. During transport and slaughter, this could include experiences such as fear, thermal discomfort, and social interactions. However, as we will discuss further in the Shape of a life and Emotional significance sections, judgments regarding the overall goodness or badness of differing welfare harms—in this case, the ones occurring at the end of life—will sometimes require additional considerations beyond just the absolute contribution of these experiences to the overall welfare. Factors such as external judgments of the ethical value and emotional significance of the harms will also come into play.

Our arguments in this paper are structured as follows. In the Shape of a life section, we discuss the relevance of the shape of a life literature to animal welfare and the significance of end-of-life concerns. In the Emotional significance section, we argue for the additional emotional significance to humans of harms occurring at the end of life, whether to animals or other humans. Finally, the Conclusion section concludes our discussion of the ethical importance of end-of-life welfare and offers some suggestions for how animal welfare during end-of-life periods can be improved.

## Shape of a Life

The ethical significance of when in an animal’s life a harm occurs relates to similar discussions on the importance of a “shape of a life” for human well-being. The shape of a life literature investigates how the impact of a harm is influenced by its context and position within a life, rather than merely the individual experience of the harm’s intensity and duration. It argues that the value of a life overall is more than the simple sum of all experiences. Instead, the distribution of experiences also matters and must be taken into account when considering the overall goodness or badness of a life. We have briefly argued in a previous paper that shape of life considerations should be an important part of thinking about humane slaughter (Browning and Veit, 2020c); arguments we will expand on here for the wider overall context of transport and slaughter. We will distinguish the different ways in which the shape of a life might be important—both internally to the animal themselves and externally from the perspective of human ethical agents.

### Instrumental value of life’s shape

The simplest way in which the shape of a life could be considered to matter is purely instrumentally—as a recognition of the fact that individuals will experience some events differently, based on what has preceded them. That is, the actual experienced intensity will be increased or decreased depending on the context. This can occur as a result of interaction effects between events—prior events creating anticipation, expectations, and memories that then influence response to future events. It might be the case, for instance, that we take the anticipation of a positive event to create more pleasure than memory of one and thus prefer to have a positive event in our future rather than our past (Slote, 1982).

This type of internal value is one that can apply to both human and animal subjects. Many animals have been shown to display behavioral signs of anticipation (Lea, 2001; Waitt and Buchanan-Smith, 2001; McGrath et al., 2016) and could thus have their welfare impacted in this way, if they are able to anticipate the upcoming negative experiences associated with transport and slaughter. Conversely, there may be some welfare benefits in having negative experiences concentrated at the end of life: if we take part of the harm of suffering as being in the ongoing effects, such as the memories of the experience. Suffering at the end of life is more temporary in this sense and does not allow time for the animal to form memories that may cause more suffering in the future. Further research into the interaction and ordering effects of animal experiences will help determine to what degree these instrumental shape of life considerations impact the overall animal welfare.

There may also be additional instrumental effects that occur from the assessment of the shape of one’s life—such as the effects of control and predictability. A human preference for particular life shapes may be, at least in part, due to a preference for control over one’s circumstances. For instance, a preference for an upward trend (as we will discuss in the following section) is often due to hard work being rewarded. Appraisal theory of emotions was primarily developed for understanding human emotions (Lazarus et al., 1970; Scherer, 1999) but has also since been shown to be applicable to other animal species. It posits that the aversiveness of an event and the negative emotional reactions associated with it can be explained through their cognitive appraisal of a number of characteristics of the situation, such as suddenness, novelty, pleasantness, predictability, and controllability (Désiré et al., 2006; Greiveldinger et al., 2009, 2011). This also appears to be true for animals other than humans, where animals show changes in behavioral and physiological responses indicative of emotional state in response to various features of situations, such as novelty (Désiré et al., 2006), suddenness (Désiré et al., 2006), predictability (Weiss 1971a; Bassett and Buchanan-Smith, 2007; Greiveldinger et al., 2007), control (Weiss 1971a, 1971b; Greiveldinger et al., 2009), and confirmation with prior expectation (Greiveldinger et al., 2011). Other work has similarly highlighted the importance of control and agency for animal welfare (e.g., Sambrook and Buchanan-Smith, 1997; Buchanan-Smith, 2011; Buchanan-Smith and Badihi, 2012; Browning and Veit, 2021; van Weeghel et al., 2021). The ability to control aversive stimuli is likely to lead to better coping ability and welfare outcomes. Thus, in part, we might explain the importance of shape of life in terms of this experience of control. It is the psychological response, rather than the physical stressor itself, that determines negative effects. Appraisal effects are likely to differ relative to the positioning of events over the lifetime and thus could form another way in which the shape of life impacts the overall quality of life. 

In these cases, it is not the shape of life itself that is valuable to the subject but rather the interaction effects that it creates between experiences. Rather than the shape of a life having its own value, it merely influences the total amount of experienced welfare across a lifetime. However, here, we wish to also explore the stronger point that even when two lives contain an equal total sum of positive and negative experiences, it is still worse to have suffering at the end of life. Regardless of the actual variation in experienced welfare, the events at the end of the life may have an additional *ethical* significance, due to their positioning. To this end, we can first consider cases where an individual—human or animal—additionally values a particular shape of life for its own sake. This appears to be particularly true when we consider the trend of change.

### The value of the trend of change

When thinking about the value of different shapes of life, humans seem to ascribe particular value to the trend of the change—whether the life quality improves or diminishes over time. It is a common intuition in thinking about human well-being that it is better to have a life that begins poorly but ends well rather than a life that begins well but ends poorly. That is, that the trend itself is valuable—an upward trend is better than a downward trend; losses are themselves bad and gains are good (Velleman, 1991; Glasgow, 2013).

This can be taken both as an internal value—where the subject of the life themselves values the trend—and as an external value, where an upward trend is taken to be an additional ethical good, regardless of the specific experiences or preferences of the subject.

The trend of change is particularly relevant to agricultural animals, given that the experience of most (if not all) of these animals is of a life that goes particularly badly at its end. Even though there may be sources of suffering and negative welfare throughout their lives, there is—as discussed in the Introduction section—a particular concentration of such experiences at the end of life, and so the overall trend tends to be a downward one. Therefore, where the shape of life considerations apply, this makes the life a worse one than if the same amount of suffering were to occur, but to ease off at the end of life such that there is an upward trend. This would thus make alleviation of end-of-life suffering particularly important.

### Internal importance of the trend of change

For humans, this value arises primarily through the narrative importance they place on the “story” of their own life—how an individual views the importance of the events in their life as shaping the overall story of their life, according to their own values and goals. This has been described as a “relational” view, in which it is the causal or narrative relationships between events that matter (Dorsey, 2015). Here, the shape of a life considerations can be intrinsically valued when the individual is able to conceive of and form a preference for their life to go a certain way, including a preference for life experiences to be distributed such that negative events do not occur at the end of life. For instance, a preference for earlier suffering followed by later pleasure can often be seen as a form of redemption. That is, that some struggle can be redeemed by contributing to an agent learning and growing in such a way that their future life is improved (Velleman, 1991; Portmore, 2007), where—importantly—the earlier suffering provided a foundation to bring about the later successes.

The intrinsic value placed on the “story” of one’s life does not seem important for the animal case, as animals are unlikely to have such meaningful relations between the events of their lives; this will be particularly true for agricultural animals that arguably lack the agency over their own experiences to make such sufferings significant in this way. Though some animals seemingly have the ability to mentally project themselves into the future (Suddendorf and Corballis, 2010), right now it appears that this ability is rare and is unlikely to result in many species—particularly those kept as agricultural animals—forming any sort of rich sense about the shape of their own life. Ongoing research into the abilities of animals in this regard will help shape the case for how strong this consideration should be for considering the importance of end-of-life welfare.

However, there is a weaker sense of the relational view in which the preference is merely for good events to follow bad ones rather than for a stronger narrative cohesion. This version of the view can still apply to humans but also seems a more plausible candidate for applying to animals. It requires only a simple structure of events improving. Glasgow (2013) gives an example of a case that demonstrates the same intuitions without requiring such a narrative trajectory. This case describes someone in the last year of their life being offered one of two drugs—one that starts out by creating severe pain that lessens over time to provide eventual bliss, or one that does the opposite, starting with bliss and giving way to pain. It seems that most would prefer the first over the second, despite the fact that both represent the same amount of total additive well-being and neither has a strong narrative or redemptive trajectory. While one might object that this can be explained by anticipation effects making the latter case worse, this is ruled out by holding fixed the total amount of pleasure or suffering. It must be the order of events themselves, rather than their additional effects, that we prefer.

The intuition is borne out in interesting experiments by Kahneman et al. (1993), who found that human subjects preferred a painful experience that ended with an “easing up” to one that was the same throughout, even though the total amount of pain experienced was greater in the former. They suggest that the peak and the end are more important than duration when retrospectively evaluating the value of an experience. Though the authors suggested that this is a failure of rationality, others have argued that this is a perfectly acceptable form of reasoning (Beardman, 2000). We simply prefer earlier pains to later ones, an upward trajectory to a downward one. We perform a retrospective evaluation of an experience, which is in part determined by the ending of the experience and shapes its value accordingly (Beardman, 2000).

This option seems potentially available to animals as well. It requires only a simple narrative structure: “a story that a dog could appreciate: it was bad, and it got better” (Beardman, 2000, p. 108). All it requires to be true of animals is that if they could, they would choose for a good event to follow a bad one rather than the other way around. Animals may also place at least some basic value on the distribution of events within their life, which is at least potentially testable. For instance, tests such as Kahneman’s experiments described above could, if modified for use in animals, provide information regarding whether different animals hold similar preferences to humans regarding the shape of unpleasant experiences. It is certainly plausible, in line with work that has demonstrated that animals appear to demonstrate frustration when experiencing shifts in life quality such that their experience is worse than expected (Greiveldinger et al., 2011). This is potentially indicative of the value placed by the animal on an upward rather than a downward trend and has led to the recommendation that “we should strive wherever possible to eliminate negative shifts in the environment of farmed or captive animals” (Greiveldinger et al., 2011, p. 814). Unfortunately, this negative trend is precisely the shift we see at the end of life for agricultural animals. By this reasoning, a life that ends with suffering would then be retrospectively evaluated as a bad life.

### External importance of the trend of change

Finally, there is the external or ethical importance of the trend of change or the overall value of a life of a particular shape. This is the value as taken from the perspective of human ethical agents judging the situation, taking some lives to be better or worse overall, independently of the specific preferences, or capacities, of the subjects of the life. It need not be the case that the individual subject of the life is able to judge their life’s overall shape and form a preference (or not), only that it would be reasonable for one performing such an evaluation on their behalf to come to such a conclusion. So, even when animals may not themselves have the capacities to conceive of and form a preference for a particular shape of life, it can still be the case that this life is in fact worse, from an external (human) ethical perspective. It may not just be the number and intensity of experiences that matter but also the way they are distributed within the life.

This can be seen in the way humans conceive of the value of the “story” of a life. A life that starts well and ends badly is taken to be a tragedy, whereas one that starts badly and ends well is a success story (Slote, 1982). It is in part our intuitions regarding these different shapes of life that shape the way we create and view fictional stories—the famous tragedies are those stories of a great fall from a good life to a poor one. We place particular importance on a life ending well. In large part, this is related to the possibility of compensation. Just as the relational view may take redemption to be an important part of the shape of a life, from an external perspective we can take compensation to be of relevantly similar importance. Thus, one of the reasons we take suffering at the end of life to be worse than at other points in time is that there is no possibility for compensating this suffering with future positive experiences. Often, we will aim to offset an experience of suffering, at least in part, by a future positive experience. This is why we often treat or reward ourselves after a stressful work situation or a painful medical procedure—it helps to in part “overwrite” the previous negative experience. We cannot compensate in the same way through prior positive experiences. Slote (1982) argues that while we readily take later gains to compensate for past harms, we do not typically take earlier successes to do the same. The obvious problem for negative end-of-life welfare is then that this sort of positive compensation does not occur.

Suffering at the end of life is unable to be compensated, and, for this reason, it is taken to be worse. It is thus in large part the way a life ends that determines the quality of goodness or badness. Due to the fact the animal ceases to exist, the impossibility of compensation at the end of life is detrimental solely from an externalist perspective but not from an internalist one (the animal’s point of view). However, as we have introduced here and will explore further in the Emotional significance section, externalist considerations form at least part of the picture of the ethical significance of the shape of life.

It is possible that such a preference comes, in part, from thinking that those with very little time left to live are less fortunate than those still with a lot of time remaining, and thus this misfortune should in some sense itself be compensated through the provision of positive experiences (Slote, 1982). If it is already a misfortune to be close to death, it is thus an additional misfortune at this time to also be suffering, in a way that it would not be for one that still has a long life ahead. It is thus, in a sense, a compensation for misfortune, where suffering at this time would instead be compounding the misfortune. Animals undergoing slaughter are particularly unfortunate in this regard—as well as the misfortunes relating to the stress and negative affects associated with the experience of transport and slaughter, they are suffering the strong misfortune of having their lives prematurely ended (Yeates, 2010; Browning, 2018; Browning and Veit, 2020c). For this reason, their need for compensation is particularly strong, and the harms occurring at the end of life correspondingly worse when such compensation is not forthcoming.

This is also compatible with thinking that earlier positive events do compensate later suffering to some degree, so long as we accept some asymmetry—that later compensation is preferable to earlier. This still makes it the case that being unable to receive subsequent compensation makes you worse off, even if you did receive earlier positive experiences that somewhat offset the suffering. Thus, while giving animals positive experiences in the earlier parts of their lives is important for overall lifetime welfare, they can not serve to fully compensate for the intensity of the negative experiences occurring during transport and slaughter.

## Emotional Significance

Another external value relevant to end-of-life welfare is its emotional significance. Here, we can draw on a discussion by Greaves (2019) of the difference between badness in the “axiological” sense and badness in the “emotional reaction” sense. Badness in the axiological sense refers to those harms that are important from a population-level or world-level stance. They are the considerations of what makes one state of affairs better or worse than another; for example, if they are greater harms occurring to a greater number. By contrast, badness in the emotional-reaction sense is those harms that are the fitting subjects of negative emotional reactions, either self- or other-regarding. These harms are important from a first-person emotional point of view, such as when they are harms occurring to ourselves or those we care about, or with a particular emotional resonance. When thinking about the added ethical importance of suffering occurring at the end of life, it is this emotional significance that also plays a strong role.

While it may be objectively no worse for any harm to occur at some specific moment within a life rather than another—as objectively we have temporal neutrality—it can be *subjectively* worse from the perspective of human ethical agents judging the situation. We may impute additional emotional harm in these cases, when considered from the external, ethical point of view. As humans, we have an even stronger negative reaction to the thought of an animal ending its life in suffering than we do to the thought of equivalent suffering occurring at some earlier time. There is some additional sense of tragedy in considering an individual’s last experiences to have been negative ones. While Greaves takes this latter form of badness as an inappropriate source of information regarding what actions we ought to take, here, we disagree. We take the emotional reaction to be an additional important consideration informing our treatment of animals and thus for investing resources into improving end-of-life welfare. This of course does not imply that we should do so if it makes the world much worse overall—if we could use the same resources to stop some much greater amount of suffering at another point in time—but simply that, all else being equal, we should pay special attention to end-of-life welfare rather than write it off as less important. There is support for this in the way we view end-of-life conditions for humans. Here, we provide two brief examples to illustrate this point: palliative care and the “last meal” for death row inmates. 

### Examples—palliative care and last meals

The emerging field of palliative care has emphasized the special importance of caring for the dying (Clark, 2007), an obvious emphasis on end-of-life welfare. Even when a life cannot be saved, it is a priority to ensure that suffering is minimized and that a high quality of life is experienced through to the end. This implies a special emotional link to the experiences at end of life. We do not want ourselves, or those we love, to suffer at the end of their lives. Again, while this may in part arise from an internal preference from individuals to have these additional moments of increased welfare, our societal emphasis on palliative care also supports the idea that there is an external ethical significance placed on end-of-life welfare. Although historically this emphasis has typically been for the human case—with veterinary medicine aiming more at quick and painless death—Selter et al. (2021) have recently argued that both professions have moved closer to each other, with veterinary palliative care and the “animal hospice” movement now also emphasizing animal quality of life rather than just painless euthanasia. This shows that our ethical judgments regarding the animal cases can be in line with the importance that we place on the human case.

Another case in which we also see concerns for end-of-life conditions is in the provision of a special “last meal” for death row inmates. Here, the circumstances are already more similar to the case of the industrial killing of animals. While prisoners usually have little choice about what food to eat, it has been common in many countries to allow someone who is sentenced to death to have the last meal for which they could order anything they want, be that something they never tasted before or their favorite foods from before entering prison (Wansink et al., 2012). This is often justified as an attempt to “make their last moments on Earth as fitting and as comforting as you can” (Graves, 2003, p. 1). Another reason for offering prisoners a final meal of their choice is an attempt to provide them with a positive experience of autonomy (LaChance, 2007), and these concerns may likewise matter to animals in captivity (Browning and Veit, 2020a, 2021). It is some sense of compassion or benevolence that motivates the desire for someone to have a pleasant experience with which to finish their lives. Though of course one may question the compassion or benevolence in the practice of execution at all, this does not mean that some of the surrounding practices cannot be motivated this way. Concern for a special last meal with which prisoners end their lives speaks to an additional ethical value placed on the welfare at the end of life.

As a society, it is clear that we place value on the provision, wherever possible, of positive experiences for those we know are at the end of their life. This appears to be motivated by an emotional resonance of the importance of this period of time for individual well-being. The time preceding death rather than simply taken as constituting the worst time of one’s life is instead a focus of attempts to improve. These arguments in favor of making the last moments of human life as good as possible make it compelling to think that they could also have the same emotional resonance when applied to animals.

### Concept of death

One may object that while these examples demonstrate the emotional significance of the end of a human life, they are not applicable to the animal case, since animals have no awareness of death or their own mortality. We have two responses: that empirical evidence challenges the assumption and that there is still sufficient reason to consider it ethically important from an external perspective.

Firstly, there is now an emerging research field (comparative thanatology) examining the responses of animals to the dead and dying as well as whether, or to what degree, animals themselves do possess a concept of death. Recent work in this area has tried to clearly lay out what features are necessary for at least a basic concept of death—such as its non-functionality and irreversibility—and to demonstrate that it is at least plausible that many animal species have the relevant capacities (Monsó, 2019; Monsó and Osuna-Mascaró, 2020). Further work in this area will help clarify exactly how animals may view their own upcoming deaths and the effects it could have on them. While this appears not to be the “rich” concept of personal mortality possessed by most humans, this does not mean that only this richer concept must be relevant for considerations of end-of-life welfare. Indeed, where many humans may lack such an understanding at the end of their lives, it would not be taken as appropriate not to treat them with the same care as anyone else. Once we abandon an anthropocentric perspective centering on the human-like experience of death, we can acknowledge the perhaps more limited recognition of death by animals and from this the relevance of their end-of-life welfare. 

Finally, as we have argued throughout, even if one thinks that end-of-life welfare does not have a special importance from a purely internal welfarist perspective, the above considerations still have an external ethical significance from the human point of view. There are other things that matter over and above those things that directly impact welfare: we can subjectively value things not directly related to our welfare or think there are things other than welfare with objective value. These same considerations can, therefore, apply to our ethical assessments of the lives of other animals.

## Conclusion

There are many types of welfare harm that animals experience during transport and slaughter. In this paper, we have argued that these harms should be weighted particularly highly because they occur at the end of life. This additional importance can arise from the internal importance arising from the experience and preferences of the individual (human or animal) subject of the life and further research into the relevant capacities and preferences of different animals, regarding how end-of-life experiences may uniquely impact their welfare, will help us better understand to what degree this holds for different animals. However, there is also the external ethical value coming from human agents evaluating the situation, particularly the emotional significance that we place on end-of-life experiences.

This of course does not mean we should ignore suffering during other life stages. There may well be a trade-off here. If we are able to use the same resources to prevent a much greater amount of suffering in another context rather than at the end of life, then we should prefer this. For example, investments into technologies aimed at improving the lives of animals may well have a greater return on investment when on a farm across the entire life of an animal rather than just specialized for during transport. However, where possible, we should make a particular effort to improve the end-of-life conditions for animals, which for agricultural animals primarily include the conditions of transport and slaughter.

This should primarily take the form of minimizing or eliminating the negative experiences associated with transport and slaughter, such as is the target of much of the current work in this area (such as Grandin and Smith, 2004; Grandin, 2010; Lines and Spence, 2014, as well as the other papers within this volume). Previously, we have detailed some suggestions to remedy this problem, such as consumer advocacy to drive industry change and campaigning for improved monitoring and enforcement of existing laws and regulations surrounding transport and slaughter (Browning and Veit, 2020c). However, it is now widely recognized that good welfare is more than not simply prevention of suffering but also the promotion of positive states (Yeates and Main, 2008; Leary and Golab, 2013; Mellor and Beausoleil, 2015). For this reason, we could also look at providing some opportunities for positive experiences at the end of life, such as pleasant sensory experiences (e.g., preferred scents or comfortable temperatures), food treats, or perhaps even investigation into the use of safe pleasure-inducing drugs that could not only help with relief of end-of-life stress but also create final positive mental states. There is plenty of research on multisensory enrichment programs that could deliver lessons on how to provide a range of pleasant experiences (e.g., Carlstead and Shepherdson, 2000; Browning-Jones and Moro, 2006; Wells, 2009), and understanding the particular species-specific sensory and cognitive capacities will also play an important role (Browning, 2019a, 2019b). For instance, recent work on pigs found positive effects of the use of enrichment during transport, such as toys, music, and scents, as well as from previous habituation to vehicle noises (Crone et al., 2021). These represent simple, practical interventions that could be used to improve end-of-life welfare during transport. Additionally, we have also discussed the importance of predictability and control in animal welfare, and interventions or changes that increase these features of the experience could also represent a welfare gain.

Finally, we acknowledge that these suggestions may appear in some way counterintuitive. There may be something seemingly insincere or distasteful about sending animals off to die while giving them little treats before they go or drugging them such that they enjoy the ride. Indeed, similar concerns have been raised against the practice of providing last meals for death row inmates—that this may downplay the seriousness of the act that is about to take place (Graves, 2003). Though these possibilities have so far been given little attention, some of the arguments in the animal ethics literature against humane slaughter and use may well apply here (Pendergrast, 2015). However, we do not take the considerations discussed in this paper to constitute a defense of animal use or slaughter but merely an exploration of the implications of their current use. That is, *if* we are using animals in agriculture, and this necessarily involves transport and slaughter, what are the special types of harms this creates, and how can they best be offset? One might also take our arguments here to strengthen the case against animal use at all, but this does not preclude a call for better treatment while the practices persist, which is precisely what we call for here. Given that animals are suffering harms at the ends of their lives, this gives us special reason to try and alleviate these harms as best we can and provide animals with the best possible end-of-life welfare.

## About the Author

**Heather Browning** is a former zookeeper turned philosopher, currently working as a postdoctoral Research Officer in animal sentience and welfare at the London School of Economics, as part of the Foundations of Animal Sentience project. She holds a PhD from The Australian National University, and her primary research interests are animal welfare, sentience, and ethics.

**Walter Veit** is a scientist and philosopher with interests stretching widely across science and philosophy. He is a PhD student at the University of Sydney. His primary research interests are located at the intersection of the *biological, social, and mind sciences* in addition to *empirically informed philosophy and ethics.* Both are reflected in the current core of his work: to move the science of consciousness to its Darwinian phase by putting the origins, function, and phylogenetic spread of animal consciousness at the center of our investigation and develop a theory of consciousness that recognizes the importance of health, ecology, and evolution. For a glimpse of his work, you can take a look at his list of publications which now includes over 40 papers.
